# Free Light Chains and Intrathecal B Cells Activity in Multiple Sclerosis: A Prospective Study and Meta-Analysis

**DOI:** 10.1155/2016/2303857

**Published:** 2016-12-28

**Authors:** Gabriella Passerini, Gloria Dalla Costa, Francesca Sangalli, Lucia Moiola, Bruno Colombo, Massimo Locatelli, Giancarlo Comi, Roberto Furlan, Vittorio Martinelli

**Affiliations:** ^1^Department of Laboratory Medicine, San Raffaele Hospital, Milan, Italy; ^2^Department of Neurology, San Raffaele Hospital, Milan, Italy; ^3^Institute of Experimental Neurology, San Raffaele Hospital, Milan, Italy

## Abstract

*Background*. The presence of CSF oligoclonal bands (OBs) is an independent prognostic factor for multiple sclerosis (MS), but the difficulties in the standardization of the test and the interlaboratory variation in reporting have contributed to its limited use in the diagnosis of the disease. Standard nephelometric assays to measure free light chains (FLC) levels have been recently developed and the test may improve the detection of intrathecal B cells activity.* Methods*. The presence of OBs, kappa and lambda FLC levels, and standard indices of intrathecal inflammation were assessed in 100 consecutive patients, including patients with MS, clinically isolated syndromes (CIS), other inflammatory diseases of the CNS, and other noninflammatory diseases.* Results*. Both KFLC and LFLC correlated strongly with the presence of OCBs and with all common tests for intrathecal inflammation (*p* < 0.001 for all comparisons). KFLC and LFLC were significantly different in patients with MS and CIS compared to the other groups (*p* < 0.001 and *p* < 0.001, resp.) and had a better diagnostic accuracy than all the other tests (area under the curve 82.3 % for KFLC index and 79.3 % for LFLC index).* Conclusion*. Nephelometric assays for KFLC in CSF reliably detect intrathecal immunoglobulin synthesis and discriminate MS patients.

## 1. Introduction

Various studies on the pathogenesis of multiple sclerosis (MS) have indicated that B cells, as the humoral component of the adaptive immune system, are active participants in the pathogenesis and lesion maintenance throughout the disease process [1]. This hypothesis has been confirmed by the positive results of recent clinical trials of anti-B cells drugs in the disease [2].

The earliest and perhaps still most consistent abnormal immunologic laboratory finding in MS is the increased concentration of Ig in the CSF and the presence of CSF-restricted oligoclonal bands (OCB), all of which constitute the pathophysiological evidences of ongoing inflammation within the CNS [3, 4]. In particular, it has been shown that the presence OCB in the CSF of patients with clinically isolated syndromes (CIS) is an independent prognostic factor for the subsequent development of the disease [5–7]. Furthermore, a more favorable long-term prognosis and slower progression of the disease in OCB-negative patients has been demonstrated [8]. OCB are IgG immunoglobulins generated by plasma blasts and plasma cells in the CSF or CNS compartment [9] and are usually detected by isoelectric focusing followed by immunoblotting.

The interpretation of results is rater-dependent, albeit quite high interrater agreement has been reported [10]. Several studies have indicated a potential diagnostic value of free light chains (FLC) in MS [11–13]. FLC, either kappa or lambda (KFLC and LFLC), are secreted by active plasma cells beside intact immunoglobulins. A standard immunonephelometric method to assess qualitative and quantitative CSF FLC levels has been recently developed, and the test has the potential to improve the detection of intrathecal B cells activity.

In this study, we aimed to validate formerly published results confirming that the KFLC and LFLC are valid biomarkers of intrathecal immunoglobulin synthesis and have a diagnostic value in CIS and MS patients.

## 2. Methods

### 2.1. Patients

Between January 1, 2014, and December 31, 2014, CSF and serum samples were collected from 100 consecutive unselected patients who were admitted to our department for a suspected neurological condition and who underwent a lumbar puncture as part of their diagnostic work-up. Patient's demographics (age at admission to hospital, gender) and clinical information (final diagnosis) were collected from hospital charts. The study was approved by Ethical Committee of San Raffaele Hospital, Milan, Italy. Written informed consent was obtained from all patients and controls.

### 2.2. Blood and CSF Samples Analyses

Paired CSF and serum samples from 100 patients were collected during diagnostic lumbar puncture and peripheral vein puncture, respectively, as standard practice. CSF and serum samples were centrifuged 10 min at 800 rpm and 10 min at 3000 rpm, respectively, and were stored at −20°C until the analyses were performed.

CSF and serum concentrations for immunoglobulins and albumin were determined within the same analytical series by immunonephelometry using a BNII System (Siemens, Germany) with calibrators and internal controls provided by Siemens, in accordance with the manufacturer's recommendations.

Quantitative expression of intrathecal total IgG synthesis was based on the CSF/serum quotients QIgG, according to different formulas. CSF indexes were defined as follows:

(i) The CSF/serum albumin quotient, Qalb = albCSF/albserum × 1000, was used to assess the blood-CSF barrier function.
(ii) Ig index (Link index) was calculated according to the Delpech and Lichtblau protein quotient QIg/Qalb (normal: 0–0,7) [14].
(iii) The absolute amount of intrathecally produced Ig (Ig_loc_) was calculated according to the Reiber-Felgenhauer Ig hyperbolic function Ig_loc_ = [QIg − Qlim(Ig)] ×  Ig_serum_ (normal: <0), with Qlim(Ig) representing the upper limit of the reference range [15].
(iv) Tourtellotte index was calculated according to the Ig synthesis rate formula [(IgCSF − Igserum/369) − (albCSF − albserum/230) × (Igserum/albserum) × 0.43)] × 5 (normal: 0–3,3) [16].

Agarose isoelectric focusing (pH 3.0–10.0) followed by immunofixation with peroxidase labelled anti-IgG antiserum was performed to detect OCB [17]. Serum samples were diluted to load the gels with equal amounts of serum and CSF total IgG (20 mg/L). The assay was carried out employing the semiautomatic instrument Hydrasys System (Sebia, France) and a designed kit (Hydragel 9 CSF Isofocusing, Sebia, France). The system can detect OCB in a concentration range of 30–125 *μ*g/L. Classification of the OCB pattern was performed according to the guidelines of an international consensus [18].

KFLC and LFLC measurement were performed employing particle-enhanced immunonephelometry using the BNII System Siemens (N Latex FLC kappa and N Latex FLC lambda kits; Siemens Healthcare Diagnostics Products GmbH, Marburg, Germany). FLC in CSF and serum samples were measured according to the manufacturer's protocol with calibrators and internal controls provided by Siemens. Serum samples were diluted 1 : 100 for KFLC and 1 : 20 for LFLC determinations; CSFs were analyzed undiluted as a starting point. According to the manufacturer, the lower detection limit for KFLC was 0.03 mg/L and for LFLC was 0.06 mg/L.

KFLC and LFLC indices were defined as quotients of KFLC and LFLC concentrations in CSF and serum divided by the respective Qalb:

KFLC index = (KFLC CSF/KFLC serum)/Qalb × 1000.

LFLC index = (LFLC CSF/LFLC serum)/Qalb × 1000.

### 2.3. Statistical Analysis

Normally distributed variables were shown as mean (SD), and differences between groups were analyzed using unpaired* t*-tests. Nonnormally distributed variables were shown as medians with 25 and 75% percentiles, and nonparametric methods (the Kruskal-Wallis and the Mann–Whitney* U* tests) were used to test for differences. Categorical variables were shown as proportions, and the differences were analyzed using *χ*^2^ tests. *p* values less than 0.05 were considered statistically significant. Receiver operator characteristic (ROC) curves were generated and the area under the curve (AUC) was calculated to compare the diagnostic accuracy of CSF indexes for predicting MS. Cut-off values with the highest accuracy were selected as the diagnostic cut-off points. Standard cut-off values for CSF indexes (Link, Tourtellotte, Reiber, and Qalb) were also tested. All statistical analyses were performed using the computing environment R (R Development Core Team, 2013).

### 2.4. Meta-Analysis

We performed a meta-analysis that incorporated results from the current study into findings from previous studies of the diagnostic accuracy of FLC for MS. We searched Pubmed and EMBASE from inception until August 1, 2016, using the search terms free light chains combined with multiple sclerosis. Reference lists of pertinent articles were reviewed to identify further relevant studies. Studies were included if they used standard nephelometric methods for the quantification of FLC levels and if data reported allowed for the calculation of the following parameters: true positives (TP), true negatives (TN), false positives (FP), and false negatives (FN). The results of the literature search are presented in a flowchart following the PRISMA guidelines.

The main outcome measure was the diagnostic test performance of the KFLC index for separating MS patients from controls (CIS, other inflammatory diseases, noninflammatory diseases), as KFLC index has been reported as the best index of intrathecal synthesis according to all studies. The following information was extracted from all studies: sensitivity (TP/(TP + FN)) and specificity (TN/(TN + FP)), names of the authors, year of publication, population characteristics (group size, percentage of inflammatory diseases in the control group, gender, and age). Data extraction was performed by two authors separately (Gabriella Passerini, Gloria Dalla Costa) to ensure accuracy and disagreements were discussed in a consensus conference. A bivariate approach with a linear mixed model has been used to estimate sensitivity and specificity across studies [19] accounting for between-study heterogeneity, and meta-regression has been performed to assess the influence of covariates on the final estimates. Meta-analysis results are presented in forest plots separately for sensitivity and specificity. All computation was performed using the R Software (R Development Core Team, 2013) with the package made [20].

## 3. Results

One hundred consecutive patients who were admitted to our department for a suspected neurological condition and who underwent a lumbar puncture as part of their diagnostic work-up have been enrolled.

According to their final diagnosis, we established several diagnostic subgroups: 34 patients fulfilled the criteria of dissemination in space and time for the diagnosis of relapsing-remitting multiple sclerosis according to latest criteria [21]; 22 patients presented a clinical isolated syndrome with typical MRI alterations but did not fulfil the diagnostic criteria; 23 patients presented with other CNS inflammatory diseases; 21 patients presented no major clinical or paraclinical sign of inflammation. Patient characteristics and patient groups are shown in Tables 1 and 2.

### 3.1. CSF Oligoclonal Bands Status and Standard Indices

CSF-restricted OCB were present in 46 patients. Patients with MS and CIS had the highest prevalence of OCB positivity (79.4% and 59%), although these percentages are lower than those previously reported in the literature [22]. Patients with noninflammatory diseases had the lowest prevalence (9.5%) of CSF OCB, as expected. The presence of OCB was significantly associated with MS or a first episode of MS (*p* < 000.1), and the test had a sensitivity of 71.4% (95% CIs: 57.8–82.7) and a specificity of 86.4% (95% CIs: 72.7–94.8).

Standard CSF indices (Qalb, Link, Tourtellotte, Reiber-Felgenhauer) were also significantly different in MS and CIS patients with respect to other inflammatory or noninflammatory CNS diseases (Table 2). The best cut-off values that maximized (sensitivity + specificity) in our sample were 0.6 for the Link index, −0.9 for the Tourtellotte index, −0.6 for the Reiber IgG synthesis rate, and 5.8 for the Qalb index. These cut-off values were similar to the reference values reported in the literature and the tests had similar sensitivities and specificities (Table 3).

### 3.2. CSF Free Lambda and Kappa Chains Levels

**Figure 1:**
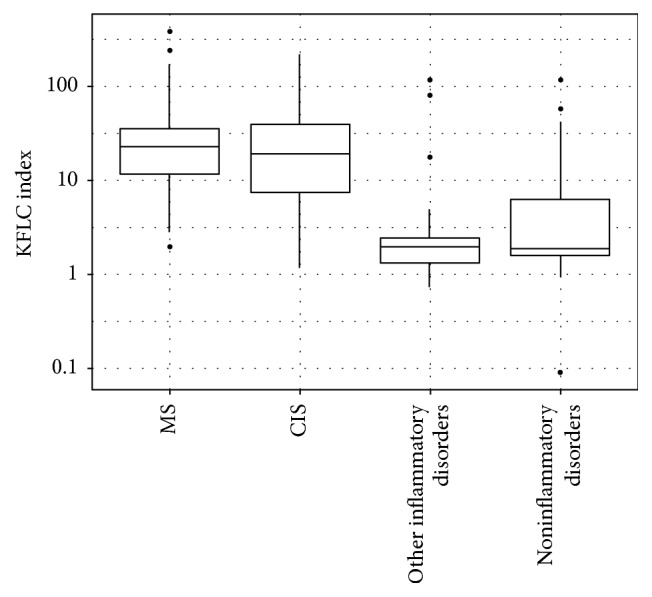
Median values and ranges of KFLC index in different subgroups: multiple sclerosis (MS) subgroup; clinically isolated syndrome (CIS) subgroup; other inflammatory disorders subgroup; noninflammatory disorders subgroup.

In patients with MS or at the onset of MS, we found high levels of both KFLC and LFLC in the CSF. There was no major difference between patients with either definite MS or a CIS. Median FLC values in CSF in MS and CIS patients were 9.1 mg/l (3.2–19.0) and 7.3 mg/l (1.4–15.2) for KFLC and 5.6 mg/l (2.2–9.3) and 4.5 mg/l (2.3–11.2) for LFLC. Patients with other inflammatory or noninflammatory diseases generally showed lower levels, both KFLC and LFLC levels in CSF (Table 4, Figure 1). All patients, including those in the MS group, exhibited FLC levels in serum within or close to the published normal ranges of healthy donors [23]. Therefore KFLC and LFLC indices were significantly different in MS and CIS patients compared to the other groups (*p* < 0.001 and *p* < 0.001, resp.). The best cut-off values that maximized (sensitivity + specificity) in our sample were 2.43 for the KFLC index and 3.04 for the LFLC index.

### 3.3. Comparison of the Diagnostic Accuracy of Free Lambda and Kappa Chains Indices to OCB and Standard CSF Indices

**Figure 2:**
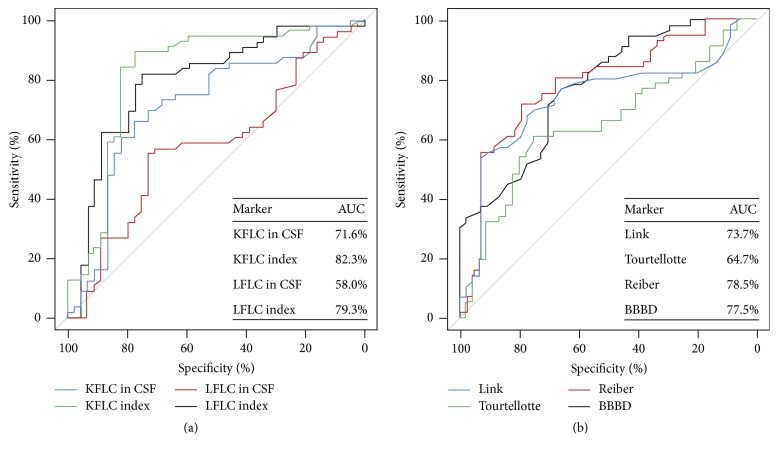
Receiver operator characteristic (ROC) curves of free light chain (a) and CSF standard indices (b) in multiple sclerosis (MS) and clinically isolated syndrome suggestive of MS diagnosis.

We compared the performance of KFLC and LFLC indices with common tests for intrathecal inflammation in their ability to discriminate MS and CIS from other neurological disorders. According to the ROC curves (Figure 2) KFLC and LFLC indices had a higher diagnostic accuracy than all the other tests, as the area under the curve (AUC) for the KFLC and LFLC indices was significantly higher than for the other tests (82.3% for KFLC index and 79.3% for LFLC index).

Sensitivities and specificities of all the tests are shown in Table 3. Compared to other indices, KFLC index was particularly good for assessing intrathecal inflammation in patients with impaired CSF-serum barrier.

**Figure 3:**
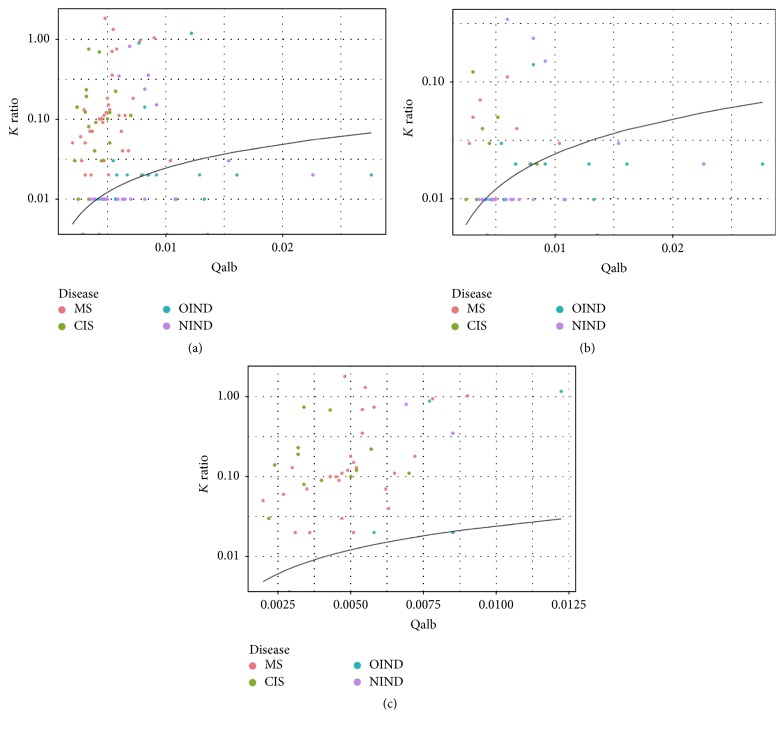
KFLC threshold line (at KFLC index 2.43) in half-logarithmic diagram with results of MS, CIS, OIND, and NIND patients (a), OCB negative patients (b), and OCB positive patients (c). CIS: clinically isolated syndrome; MS: multiple sclerosis; OIND: other inflammatory neurological disease; NIND: noninflammatory neurological disease; KFLC: kappa free light chain; OCB: oligoclonal bands.

Positive OCB correlated strongly with both KFLC and LFLC indices. Out of 46 patients with detectable OCB, 44 presented with elevated KFLC index (*p* < 0.001) and 36 with elevated LFLC index (*p* < 0.001). There was high correlation also between LFLC and KFLC indices and common tests for intrathecal inflammation (*p* < 0.001 for all comparisons). KFLC index was helpful in the discrimination of MS and CIS patients from patients with other diseases, particularly in patients with negative OCB (Figure 3).

### 3.4. Meta-Analysis

**Figure 4:**
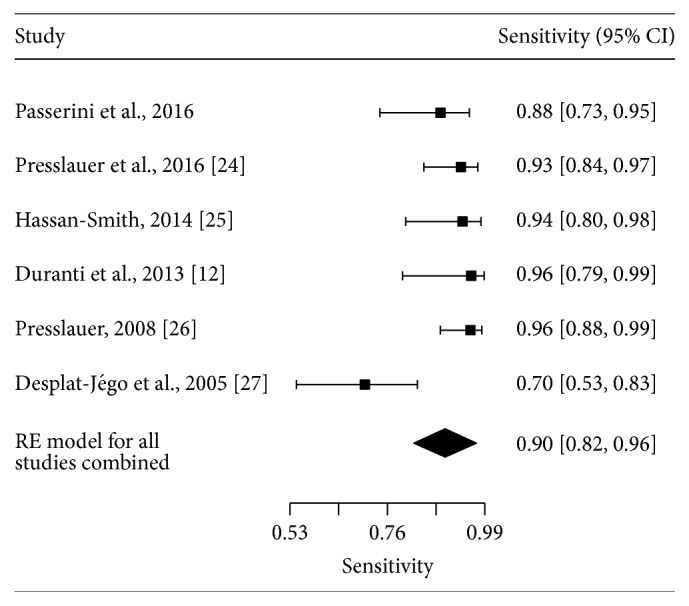
Forest plot of sensitivities for studies using KFLC index to diagnose MS. Summary estimate for sensitivity is computed using the approach described by Reitsma et al.

**Figure 5:**
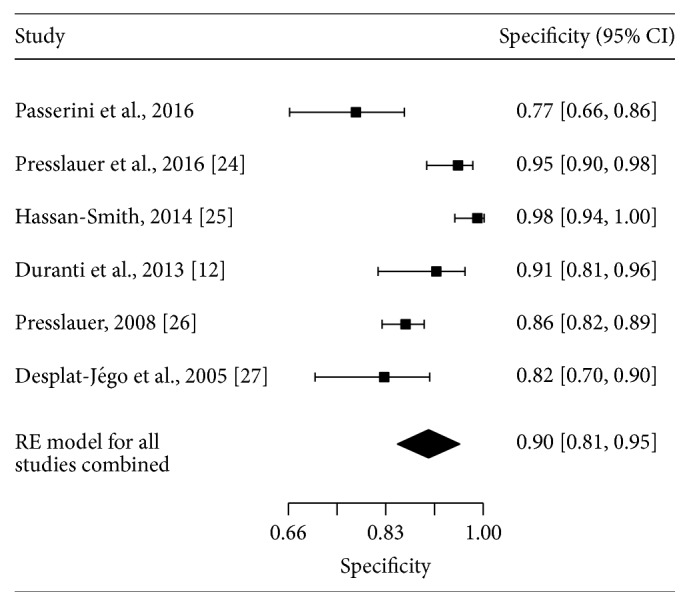
Forest plot of specificities for studies using KFLC index to diagnose MS. Summary estimate for specificity is computed using the approach described by Reitsma et al.

**Figure 6:**
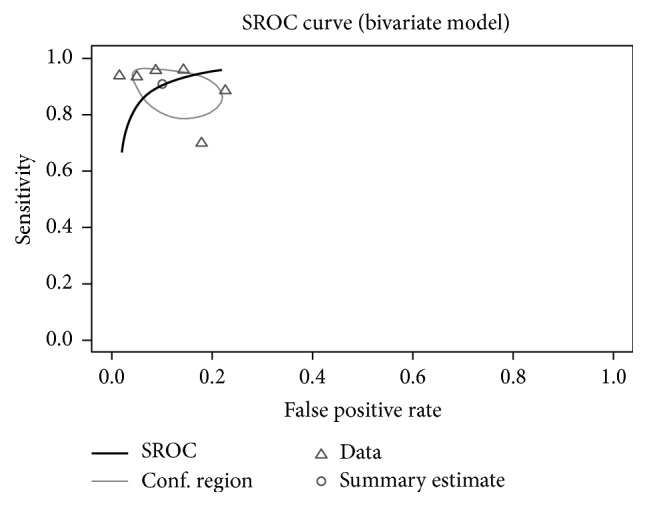
SROC curve of the Reitsma model with the summary estimated (circle) and its confidence interval (elliptic).

The initial literature search identified 95 studies of interest. After screening all studies and applying the inclusion criteria, five studies have been identified [12, 24–27]. Together with the current study, six studies with a total of 252 MS cases ascertained among 1047 adults were included in the meta-analysis. Across all studies, KFLC index spared MS cases from controls with a sensitivity of 90.1% (95% CI: 81.6–95.6%; see Figure 4) and a specificity of 89.9% (95% CI: 80.8–95.0%; see Figure 5). A summary ROC curve of the included studies along with the estimated summary is presented in Figure 6. Regression on mean age of the population, female : male ratio, percentage of patients with inflammatory disease among the controls, and cut-off value did not show any effect on sensitivity or specificity.

## 4. Discussion

Increasing evidence suggests that B cells, as the humoral component of the adaptive immune system, are active participants in the pathogenesis of MS and lesion maintenance throughout the disease process. OCB, immunoglobulins generated by plasma blasts and plasma cells in the CSF or CNS compartment [9], have long been considered the gold standard sign of intrathecal inflammation, and their presence has been shown to be an independent prognostic factor in CIS patients [5–7] and associated with a more favorable long-term prognosis in MS patients [8]. The rater-dependent interpretation of the results and the moderate diagnostic sensitivity in patients with CIS have contributed to its limited use in the diagnosis of multiple sclerosis. FLC have been previously reported as surrogate markers of intrathecal immunoglobulin synthesis, but the test is not actually employed into diagnostic use due to the fact that the determination of FLC was technically difficult in the past and not feasible in clinical routine. Recently, novel automated assays for FLC determination have been introduced.

We applied here a standard immunonephelometric method to assess qualitative and quantitative CSF FLC levels, and our results show that FLC levels, particularly KFLC index, have a good diagnostic accuracy for MS. KFLC index well correlates with OCB status and seems to have a superior diagnostic accuracy compared with common indices of intrathecal synthesis, particularly in case of CSF-blood brain barrier damage. The significant correlation of KFLC index with OCB status supports observations from other studies that suggest CSF KFLC to be elevated in patients with intrathecal IgG synthesis, independently of the type of clonality. According to our results, KFLC allow the discrimination of MS-CIS patients from patients with other diseases, particularly in patients with negative OCB. Our results are consistent with recent reports on the use of such assays for the detection of FLC and validate their use for routine detection of intrathecal immunoglobulin synthesis [11–13]. Notably, comparability of FLC thresholds used among different studies is low due to differences in the study design and population and differences in the threshold selection methods. It would be thus worthwhile to assess the contribution that KFLC index could provide to MRI biomarkers of MS currently used in the diagnostic criteria and evaluate a cut-off value that would maximize the discrimination improvement provided by the KFLC index. Additionally, these findings underline the relevance of CSF parameters in MS and CIS. In fact, despite CSF analysis no longer being a fundamental examination for the diagnosis of RRMS, CSF should be analyzed in CIS patients, given that the evidence of intrathecal synthesis is a supportive factor for an accurate diagnosis of MS, may have potential prognostic value, and may be helpful for clinical and therapeutic decision-making.

Overall, presenting at least equal diagnostic accuracy KFLC determination has the potential to replace OCB during diagnostic work-up in suspected demyelinating CNS diseases. The nephelometric assay for the detection of FLC is methodologically easy to perform and to standardize and it is rapid, and as such it would be easily integrated into laboratory processes. Furthermore, interpretation of KFLC is unequivocal as it provides a quantitative measure and would allow easy following of changes in intrathecal immunoglobulin synthesis.

Further, multicentric prospective studies enrolling a large number of CIS patients are necessary in order to evaluate the discrimination improvement offered by FLC to current MRI criteria and, ultimately, the utility of FLC in the diagnosis of MS.

## Figures and Tables

**Table 1:** Characteristics of the patient groups.

	All patients^a^ (*n* = 100)	Subgroup^b^
Group 1: MS	34 (34)	
Group 2: CIS	22 (22)	
Group 3: Other inflammatory diseases	23 (23)	
Dysimmune leukoencephalopathy		10 (43.5)
Meningitis, encephalitis		2 (8.6)
Cranial neuritis		3 (13.0)
Polyneuropathy		5 (21.7)
Vasculitis		1 (4.3)
Behcet's disease		1 (4.3)
Neuromyelitis optica		1 (4.3)
Group 4: Noninflammatory diseases	21 (21)	
Neurodegenerative diseases		6 (28.6)
Cerebrovascular diseases		4 (19.0)
Neoplastic diseases		3 (14.3)
Metabolic encephalopathy		6 (28.6)
Migraine		1 (4.8)
Psychiatric disorders		1 (4.8)

^a^Expressed as number (%) of all patients.

^b^Expressed as number (%) of the rows' total.

**Table 2:** Demographics and standard CSF characteristics of the patient groups.

Characteristic	MS (*n* = 34)	CIS (*n* = 22)	Other inflammatory diseases (*n* = 23)	Noninflammatory diseases (*n* = 21)	*p*
Age, median (IQ range)	37.4 (27.8–46.3)	28.5 (24.4–43.0)	53.0 (46.8–64.7)	46.9 (41.8–62.9)	<0.001
Gender					
Females, number (%)	21 (61.8)	17 (77.3)	12 (52.2)	15 (71.4)	ns
Males, number (%)	13 (38.2)	5 (22.7)	11 (47.8)	6 (28.6)	
CSF-restricted oligoclonal bands					
Negative, number (%)	7 (20.6)	9 (41.0)	19 (82.6)	19 (90.5)	<0.001
Positive, number (%)	27 (79.4)	13 (59.0)	4 (17.4)	2 (9.5)	
Link index, median (IQ range)	0.6 (0.6–0.8)	0.6 (0.4–0.7)	0.5 (0.4–0.5)	0.5 (0.5–0.6)	<0.001
Tourtellotte index, median (IQ range)	1.7 (−2.2–5.2)	−1.7 (−4.5–1.1)	−3.0 (−3.8–0.7)	−1.9 (−2.8–0.9)	<0.001
Reiber IgG, median (IQ range)	−0.1 (−0.5–0.7)	−0.4 (−1.1–0.0)	−1.0 (−1.9–0.5)	−1.0 (−1.7–0.7)	<0.001
Qalb, median (IQ range)	5.1 (4.1–6.2)	4.2 (3.4–5.2)	7.0 (5.0–9.9)	7.1 (5.2–9.8)	<0.001

**Table 3:** Diagnostic accuracy of free light chain (a) and CSF standard indices (b) in multiple sclerosis (MS) and clinically isolated syndrome suggestive of MS diagnosis.

Markers	Cut-off point	Sensitivity	Specificity	Positive LR	Negative LR
KFLC (mg/l) in CSF	4.1	66.1	77.3	2.9	0.4
KFLC index	2.4	89.3	77.3	3.9	0.1
LFLC (mg/l) in CSF	4.3	55.4	72.7	2.0	0.6
LFLC index	3.0	82.1	75.0	3.3	0.2
Link index	0.6	53.6	93.2	7.9	0.5
Tourtellotte index	−0.9	60.7	75.0	2.4	0.5
Reiber IgG	−0.6	71.4	79.5	3.5	0.4
Qalb index	5.8	76.8	65.9	2.3	0.4

KFLC, kappa free light chain; LFLC, lambda free light chain; LR, likelihood ratio.

**Table 4:** Comparison of free light chains levels in CSF and free light chain indices in different patient groups.

Characteristic	MS (*n* = 34)	CIS (*n* = 22)	Other inflammatory diseases (*n* = 23)	Noninflammatory diseases (*n* = 21)	*p*
KFLC (mg/l) in CSF, median (IQ range)	9.1 (3.2–19.0)	7.3 (1.4–15.2)	2.3 (1.1–3.4)	1.3 (1.1–4.0)	0.002
KFLC index, median (IQ range)	22.4 (11.4–34.9)	17.4 (3.7–34.8)	1.9 (1.3–2.4)	1.8 (1.5–2.2)	<0.001
LFLC (mg/l) in CSF, median (IQ range)	5.6 (2.2–9.3)	4.5 (2.3–11.2)	3.2 (1.1–5.7)	4.7 (2.1–6.2)	0.56
LFLC index, median (IQ range)	7.5 (3.5–12.9)	5.9 (3.8–16.0)	2.5 (1.6–2.9)	2.8 (1.7–4.1)	<0.001

KFLC, kappa free light chain; LFLC, lambda free light chain; LR, likelihood ratio.
